# Supplementary material for: Trained Innate Immunity in Animal Models of Cardiovascular Diseases
Source: Int J Mol Sci. 2024 Feb 15;25(4):2312. doi: 10.3390/ijms25042312 (PMC10889825; doi:10.3390/ijms25042312)
Supplement: Supplementary file 1 [file ijms-25-02312-s001.zip › ijms-2848478-supplementary.pdf]

Supplementary Table S1. Selection of pathogens and pathogen-derived vaccines that elicit a trained immunity effect.

Pathogen Species	Pathogens (1st Stimulus)	Host Species	Cell Type	Biological/Immunological Effects	References
BACTERIA	BCG vaccine	Human and mice	Monocytes<br>Macrophages<br>NK-cells	• Induction of non-specific protection against various infections beyond tuberculosis, such as yellow fever and malaria, e.g., due to enhanced cytokine production upon a 2nd stimulus.	[24,25,26]
	Salmonella typhii vaccine	Human	Monocytes	• Oral vaccination with attenuated Salmonella typhii causes a reaction to a second non-specific stimulus with increased cytokine production, and enhanced expression of TLR4/5, as well as multiple surface markers on monocytes.	[27]
	Pseudomonas aeruginosa	Human	Epithelial cells	• Exposure of respiratory epithelial cells with P. aeruginosa modulated their response to a subsequent different stimulus.<br>• Employing inhibitors of histone acetyltransferase and methyltransferase, epigenetic regulation was assumed as the reason.	[16]
	Acinetobacter baumannii	Mouse	Macrophages	• Intranasal vaccination can induce a training effect in alveolar macrophages and protected against subsequent infection not only with A. baumanii but also with P. aeruginosa.<br>• Enhanced TLR4- and TNF-α expression was observed in RAG-1 deficient mice.	[17]
	Neisseria gonorrhoe	Human	Macrophages	• Epigenetic modifications, like enriched H3K9ac, were detetected, indicating that N. gonorrhoe leads to a trained immunity phenotype in macrophages.	[18]
VIRUSES	Measles vaccine	Human		• Mortality studies in developing countries suggest that the measles vaccine may provide a broad general positive impact on mortality	[28]

				rates, independent of its targeted protection against measles.	
	Smallpox vaccine	Human		• Immunization correlated with improved long-term survival in a high-income country, not accountable for by the vaccine's specific protective effects.	[29]
	Oral polio vaccine (OPV)	Human		• Vaccination with OPV reduces all course child-hood mortality in developing-countries.	[30]
	Influenza vaccine	Human	Monocytes	• Influenza vaccination was linked to a reduction in SARS-CoV-2 infections and an improved immune response concerning various viral stimuli.<br>• In addition to adaptation in surface molecules of monocytes, decreased production of Il-1ß and IL-6 and additionally an increase in IL-1-RA could be shown.	[31]
	Herpesvirus	Mice	Macrophages	• Protection against a subsequent infection with Listeria monocytogenes and Yersinia pestis due to enhanced production of IFN- γ and activation of macrophages.	[19]
	Hepatitis B virus	Human	Monocytes	• Exposure of newborns to HBV in utero appears to trigger a state of trained immunity.<br>• In vitro analyses revealed that umbilical cord blood exhibited heightened responsiveness to bacterial infection, evidenced by alterations in cytokine production, for instance.	[20]
FUNGI	*Candida albicans*	Mouse	Monocytes	• ß-glucan in fungal cell wall triggers alterations in the genetic expression of immune-related genes, leading to heightened cytokine production in monocytes.	[21]
	Cryptococcus neoformans	Mouse	Dendritic cells	• Enhanced proinflammatory cytokine production, such as IFN-γ, associated with	[22]

				epigenetic changes in response to a 2nd stimulus.	
PARASITES	*Plasmodium falciparum*	Human	PBMCs	• After exposure to Plasmodium falciparum, the response of PBMCs was increased when exposed to TLR2 stimulation. Characteristic epigenetic and metabolic alterations associated with a trained immunity phenotype have been identified.	[23]
